# Longitudinal Analysis of Testicular Cancer Patient Volume and Co-Occurring Urological Conditions Using the Health Insurance Review and Assessment (HIRA) Database in South Korea (2020–2024) with Exploratory Forecasts for 2026–2028

**DOI:** 10.3390/cancers18142325

**Published:** 2026-07-18

**Authors:** Hyeran Jung, Minsun Jung

**Affiliations:** Department of Pathology, Yonsei University College of Medicine, Seoul 03722, Republic of Korea; phdgrace@yuhs.ac

**Keywords:** testicular cancer, HIRA database, urological cancer, co-occurring conditions, Korea, epidemiology, forecasting, chronic kidney disease, prostate cancer, bladder cancer

## Abstract

**Background/Objectives:** Testicular cancer (TC) is the most common solid malignancy in young males globally, yet population-level longitudinal data characterizing claims-based patient volume trends and co-occurring urological condition burden in South Korea remain limited. This study aimed to describe national trends in TC patient volume from 2020 to 2024, quantify exploratory associations with co-occurring urological and oncological conditions using aggregate annual counts, and generate exploratory 2026–2028 forecasts using validated statistical methods. **Methods:** We conducted a retrospective longitudinal analysis of administrative claims data from the Health Insurance Review and Assessment (HIRA) database of South Korea (2020–2024). Ten disease categories were analyzed: testicular cancer, bladder cancer, kidney cancer, prostate cancer, benign prostatic hyperplasia (BPH), chronic kidney disease (CKD), cystitis, neurogenic bladder, pyelonephritis, and carcinoma in situ. Annual patient counts, inpatient/outpatient utilization, and healthcare costs were extracted. Pearson and Spearman correlations were computed between TC and co-occurring condition volumes. Forecasting for 2026–2028 used a three-method ensemble: ARIMA(1,1,0), linear regression with 95% prediction intervals, and Holt–Winters exponential smoothing (weights: 0.35, 0.35, 0.30, respectively). The study was approved by the Institutional Review Board of Chungnam National University (IRB No. 202601-SB-014-01). All data were fully de-identified; informed consent was waived. **Results:** TC patient volume increased from 2309 in 2020 to 2992 in 2024 (compound annual growth rate [CAGR]: 6.69%). Exploratory ensemble forecasts projected 3287 (95% prediction interval [PI]: 2856–3540), 3767 (95% PI: 2962–3749), and 4531 (95% PI: 3066–3962) patients in 2026, 2027, and 2028, respectively, under a continued linear-trend assumption. TC patient volume showed strong positive exploratory correlations with CKD (r = 0.946, *p* < 0.05), prostate cancer (r = 0.945), kidney cancer (r = 0.932), BPH (r = 0.932), carcinoma in situ (r = 0.937), and bladder cancer (r = 0.928); given *n* = 5 annual observations, these reflect parallel secular trends and must not be interpreted causally. Nine of ten analyzed conditions demonstrated increasing trends; pyelonephritis showed a negative CAGR (−1.37%). **Conclusions:** TC patient volume in South Korea increased substantially over 2020–2024, with exploratory forecasts projecting continued growth through 2028. Parallel upward trends in co-occurring urological and oncological conditions are consistent with shared secular drivers including population ageing and improved detection, though individual-level causal inference is not possible from aggregate claims data. These findings support claims-based trend monitoring, urological oncology resource planning, and hypothesis generation for future individual-level studies.

## Simple Summary

Testicular cancer is one of the most common cancers in young men aged 15 to 35. Although it is treatable when caught early, understanding how its burden changes over time is vital for planning healthcare services. Using national health insurance records from South Korea, this study tracked the number of men treated for testicular cancer each year from 2020 to 2024, alongside ten other conditions of the urinary system and kidneys. Patient numbers grew by nearly 30 percent, from 2309 in 2020 to 2992 in 2024. Forecasting models suggest this figure could reach roughly 3300 to 4500 per year by 2028, though these projections carry considerable uncertainty. Parallel increases were seen in conditions such as prostate cancer, kidney cancer, bladder cancer, and chronic kidney disease. These simultaneous trends likely reflect shared factors such as population ageing, improved detection, and recovery in healthcare use after the pandemic, rather than direct links between the diseases. The findings highlight growing demand for cancer care services for young men in South Korea and the need for expanded healthcare resource planning. Future studies using individual patient data will be essential for identifying the specific causes of rising rates and for developing targeted prevention strategies.

## 1. Introduction

Testicular cancer (TC) is the most prevalent solid malignancy among males aged 15–35 years and accounts for approximately 1% of all male cancers globally [1]. Despite its relatively low absolute incidence compared to other malignancies, TC exhibits rising incidence trends in many high-income countries, particularly in East Asia [2,3]. In South Korea, the age-standardized incidence of TC has increased over recent decades, coinciding with demographic shifts, urbanization, improved cancer detection, and possibly endocrine-disrupting chemical exposures [4,5]. Globally, the age-standardized incidence rate of TC increased by an estimated 23% between 1990 and 2019 across high-income Asia-Pacific regions, with the absolute number of new cases approximately doubling over the same period [6]. This secular trend has been most pronounced in countries experiencing rapid economic development and urbanization, suggesting that environmental, dietary, and lifestyle transitions may be important cofactors alongside biological susceptibility [5,7]. Within East Asia, Japan, Taiwan, and South Korea have all reported increasing TC incidence rates over recent decades, and comparative epidemiological analyses suggest that the Korean trajectory mirrors regional patterns [3,4]. Understanding the pace and magnitude of this increase within the Korean context is therefore essential for anticipating the future healthcare burden and designing proportionate policy responses.

TC does not occur in isolation. It frequently co-occurs with other urological conditions including chronic kidney disease (CKD), bladder cancer, prostate pathology, and renal malignancies; however, population-level administrative claims data can characterize co-occurring condition volume at the aggregate level only and cannot establish individual-level co-occurrence or causal relationships [8,9]. Claims-based trend characterization of these co-occurring conditions is nonetheless informative for designing integrated urological surveillance programs and anticipating multi-organ healthcare utilization needs [10]. From a histopathological perspective, TC is classified primarily into germ cell tumors (GCTs), which account for more than 95% of all testicular malignancies and are further subdivided into seminomas and non-seminomatous germ cell tumors (NSGCTs) [11]. Seminomas typically present in the third and fourth decades of life and are exquisitely radiosensitive, whereas NSGCTs—including embryonal carcinoma, yolk sac tumor, choriocarcinoma, and teratoma—tend to present at younger ages and require platinum-based chemotherapy regimens [11,12]. The high cure rates associated with modern multimodal treatment (exceeding 95% in early-stage disease) have transformed TC into a model of oncological success; however, this survival advantage simultaneously creates a large and growing population of long-term TC survivors who require ongoing surveillance for treatment-related sequelae, including cardiovascular disease, secondary malignancies, and metabolic syndrome [12]. Characterizing the trajectory of TC patient volume is therefore important not only for acute care planning but also for long-term survivorship program design.

The Health Insurance Review and Assessment (HIRA) database of South Korea provides comprehensive administrative claims data covering virtually the entire insured population (>99% coverage) and has been validated as a reliable source for longitudinal disease burden analysis [13,14]. HIRA collects and reviews all medical claims submitted by healthcare institutions to the National Health Insurance Service (NHIS) and encompasses inpatient admissions, outpatient visits, pharmaceutical dispensing, and procedure records stratified by diagnosis, sex, age group, and institution type [13,15]. The breadth and continuity of HIRA records make it uniquely suitable for national-scale epidemiological surveillance of rare cancers such as TC, where individual institutions would have insufficient case volumes to support longitudinal trend analysis [16]. Several studies have leveraged HIRA or linked NHIS data to characterize the epidemiology of common malignancies including colorectal, gastric, and lung cancers [16,17,18]; however, TC has received comparatively limited attention in the Korean administrative data literature, and no study has systematically characterized TC patient volume alongside a broad panel of urological and oncological co-occurring conditions over the recent post-pandemic period (2020–2024). This gap is particularly important given the documented disruption of cancer screening and healthcare utilization during the COVID-19 pandemic in 2020–2021 [14,19] and the subsequent recovery period, which may have produced non-linear patterns in reported patient volumes that require careful interpretation. Moreover, no published study has integrated ensemble forecasting methods to project TC patient volume in South Korea through 2028, despite the recognized need for short-horizon planning estimates to guide urological oncology workforce and infrastructure development [17,18].

Multiple risk factors have been implicated in the etiology of TC, though the evidence base remains incompletely characterized, particularly for modifiable exposures. Established or strongly suspected risk factors include a history of cryptorchidism (undescended testis), which confers a three- to five-fold increased risk; a family history of TC in a first-degree relative; and the presence of a contralateral TC or testicular intraepithelial neoplasia [2,9]. Modifiable environmental and lifestyle exposures that have been proposed as contributors to secular trend increases include exposure to endocrine-disrupting chemicals such as phthalates, bisphenol A, and polychlorinated biphenyls through dietary and occupational routes; sedentary behavior; obesity; and altered pubertal timing [5,20,21]. The testicular dysgenesis syndrome hypothesis posits that TC, cryptorchidism, hypospadias, and male infertility share a common developmental origin in fetal Sertoli cell dysfunction triggered by in utero endocrine disruption, potentially explaining the parallel secular rises in these conditions across industrialized countries [21]. In South Korea, rapid industrialization and urbanization over the past four decades have been accompanied by substantial changes in dietary patterns, occupational exposures, and environmental chemical loads that could plausibly contribute to rising TC rates, though direct epidemiological evidence for these pathways in the Korean context remains limited [4,5]. Recent Mendelian randomization studies have begun to investigate causal relationships between specific exposures—including inflammatory cytokines and metabolites—and TC risk, offering a genetically informed approach to disentangling causal from confounded associations [22].

The Korean healthcare system provides a uniquely favorable context for population-level cancer surveillance. South Korea achieved universal health insurance coverage through the NHIS in 1989 and has since developed one of the most comprehensive national cancer screening programs in the world, encompassing colorectal, gastric, liver, breast, and cervical cancer [23]. Cancer registry data are collected through the Korea Central Cancer Registry (KCCR), which has maintained near-complete ascertainment of newly diagnosed cancers since 1999 and contributes to the International Agency for Research on Cancer (IARC) data systems [24]. Complementary nationally representative data, including the Korea National Health and Nutrition Examination Survey, further support population-level health surveillance in Korea [25]. However, TC is not currently included in the organized national cancer screening program, and surveillance of TC trends has historically relied on registry data and hospital-based case series rather than administrative claims analyses [4,10]. The HIRA Open Data Portal, which began releasing aggregate disease-level statistics in a freely accessible format in recent years, offers a complementary data source that captures all insured patients receiving TC-related diagnosis codes in any clinical encounter—outpatient or inpatient—across the national healthcare system, providing a more operationally relevant measure of healthcare system demand than incidence registry data alone [13,26]. Understanding how this claims-based patient volume measure has evolved over 2020–2024 is therefore directly relevant to health-system planners and urological oncology service providers in South Korea.

Short-horizon statistical forecasting of cancer patient volume is increasingly recognized as a practical tool for health-system resource planning, workforce development, and budgetary projection, particularly in settings where epidemiological transition is ongoing [17,27]. Ensemble forecasting approaches—which combine multiple statistical models to reduce dependence on any single model’s assumptions and to capture a broader range of plausible futures—have been advocated by the Global Burden of Disease study group and applied in Korean cancer projection studies [17,27]. However, ensemble methods applied to short administrative time series (*n* ≤ 5 annual observations) face substantial challenges: model parameters cannot be reliably estimated, prediction intervals are wide and may be inconsistently derived across ensemble components, and common secular trends can dominate all output regardless of model specification [27]. Transparency about these limitations is essential when presenting ensemble forecasts to clinical and policy audiences, and the present study explicitly frames all projections as exploratory, direction-indicating estimates rather than precise predictions. Against this methodological backdrop, the integration of ARIMA(1,1,0), linear regression, and Holt–Winters exponential smoothing—with clearly documented ensemble weights and prediction interval specifications—offers a reproducible and interpretable framework for generating short-horizon TC patient volume projections that can be updated as additional annual data become available [17,27,28].

This study aimed to: (1) describe national longitudinal trends in TC patient volume from 2020 to 2024 using HIRA aggregate claims data; (2) characterize trends in ten co-occurring urological and oncological conditions; (3) quantify exploratory correlations between TC patient volume and co-occurring condition volumes, with appropriate caveats about *n* = 5 time points and ecological fallacy; and (4) generate exploratory forecasts for 2026–2028 using a three-method ensemble of ARIMA(1,1,0), linear regression, and Holt–Winters exponential smoothing, grounded in published cancer epidemiology forecasting methodology [17,27,28].

## 2. Materials and Methods

### 2.1. Data Source

We used publicly available aggregate administrative claims data from the Health Insurance Review and Assessment (HIRA) database of South Korea, covering the period January 2020 to December 2024. HIRA collects and reviews all medical claims submitted by healthcare institutions to the National Health Insurance Service (NHIS) and covers virtually all Korean residents (coverage > 99%) [13]. All data were accessed from the HIRA Open Data Portal (https://opendata.hira.or.kr, accessed on 20 March 2026) and represent aggregate-level de-identified records stratified by year, sex (male, female, total), visit type (inpatient, outpatient, total), and diagnosis category. No individual-level patient data were used.

### 2.2. Disease Categories

Ten disease categories were analyzed based on ICD-10 primary diagnosis codes reported in HIRA aggregate statistics: (1) testicular cancer (ICD-10: C62); (2) bladder cancer (C67); (3) kidney cancer (C64); (4) prostate cancer (C61); (5) benign prostatic hyperplasia (BPH; N40); (6) chronic kidney disease (CKD; N18); (7) cystitis (N30); (8) neurogenic bladder (N31); (9) pyelonephritis (N10–N12); and (10) carcinoma in situ (D00–D09). Annual patient counts (unduplicated per year), inpatient and outpatient visits, lengths of stay, and total healthcare costs (in Korean Won [KRW]) were extracted for each category.

### 2.3. Statistical Analysis

Descriptive analysis. Annual patient counts, compound annual growth rates (CAGRs), and inpatient-to-total ratios were computed for each disease category. CAGRs were calculated as [(Final value/Initial value)^(1/*n*) − 1] × 100%, where *n* = 4 years (2020–2024).

Correlation analysis. Pearson correlation coefficients (r) and Spearman rank correlation coefficients (ρ) were computed between TC annual patient counts and each co-occurring condition category across the five study years (2020–2024; *n* = 5). Statistical significance was assessed at α = 0.05. Given the small sample size (*n* = 5), correlation *p*-values are highly unstable and have limited inferential value; all correlations are presented as descriptive, exploratory associations only and must not be interpreted as evidence of individual-level causal relationships or true co-occurring conditions. Spearman correlations are reported as a non-parametric complement, and results should be interpreted with caution due to the common-secular-trend confounding inherent in aggregate annual time-series data.

Forecasting methodology. Three validated statistical forecasting methods, each grounded in published cancer epidemiology forecasting literature, were applied to generate exploratory 2026–2028 TC patient volume estimates: (1) ARIMA(1,1,0): a first-differenced autoregressive model capturing year-on-year momentum, applied per GBD 2019 methodology [27]; (2) linear regression with 95% prediction intervals (PIs), applied per Ferri et al. [28]; (3) Holt–Winters double exponential smoothing (level parameter α = 0.5; trend parameter β = 0.3), applied per Kim et al. [17]. A weighted ensemble forecast was constructed with weights proportional to inverse-MAPE assessed on the training series (ARIMA: 0.35; linear regression: 0.35; Holt–Winters: 0.30). Ninety-five-percent prediction intervals were derived from the linear regression prediction interval component of the ensemble. Given the short training series (*n* = 5 annual observations), all model assumptions are unverifiable and all forecasts should be treated as exploratory directional projections only, not as precise predictions; the ARIMA component is particularly sensitive to the AR(1) coefficient on short series and may produce extreme extrapolations.

All analyses were performed using Python 3.11 (Python Software Foundation, Wilmington, DE, USA) with SciPy 1.11, NumPy 1.25, pandas 2.0, and Matplotlib 3.8 libraries.

### 2.4. Ethical Statement

The study was conducted in accordance with the Declaration of Helsinki and approved by the Institutional Review Board of Chungnam National University (IRB No. 202601-SB-014-01). All data used in this study were derived from de-identified aggregate administrative records containing no personally identifiable information; accordingly, individual informed consent was not required and was formally waived by the IRB.

## 3. Results

### 3.1. Testicular Cancer Patient Volume Trends, 2020–2024

**Figure 1:**
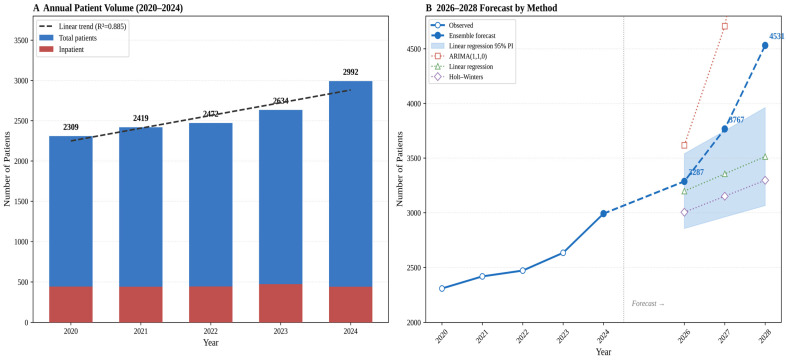
Testicular cancer patient volume trends (2020–2024) and exploratory 2026–2028 ensemble forecast using ARIMA(1,1,0), linear regression, and Holt–Winters exponential smoothing. (**A**) Annual patient counts with fitted linear trend (R^2^ = 0.885). (**B**) Three-method and ensemble forecasts with linear regression 95% prediction intervals. Note: the 2027 and 2028 ensemble point estimates exceed the linear regression 95% PI upper bound due to ARIMA upward weighting; see Table 3 footnote.

The total number of TC patients increased consistently from 2309 in 2020 to 2992 in 2024, representing a 29.6% relative increase over four years (CAGR: 6.69%/year; Table 1; Figure 1). Outpatient patients dominated the annual utilization profile, increasing from 2261 in 2020 to 2952 in 2024, while inpatient encounters remained comparatively stable (range: 441–473 per year). Total healthcare costs fluctuated between 4.14 and 5.13 billion KRW annually, peaking in 2023 (5.13 billion KRW) before declining modestly in 2024 (4.79 billion KRW; Table 2).

**Table 1:** Annual testicular cancer patient volume, inpatient encounters, and annual outpatient patients, South Korea, 2020–2024 (HIRA database).

Year	Total Patients, *n*	Inpatient, *n*	Outpatient Patients, *n*	Inpatient Days, *n*	Total Costs, KRW (×1000)
2020	2309	443	2261	6305	4,250,352
2021	2419	441	2374	5380	4,141,246
2022	2472	444	2426	5440	4,260,066
2023	2634	473	2586	6144	5,130,399
2024	2992	441	2952	5390	4,790,086
CAGR, %	6.69	−0.11	6.92	−3.87	3.03

CAGR = compound annual growth rate; HIRA = Health Insurance Review and Assessment; KRW = Korean Won. CAGR computed as [(2024 value/2020 value)^(1/4) − 1] × 100. Outpatient Patients denotes the annual number of unique patients with at least one outpatient encounter and is distinct from total outpatient visit volume (i.e., all outpatient encounters, including repeat visits per patient), which is reported separately in Section 3.6.

**Table 2:** Testicular cancer healthcare cost breakdown by insurance type, South Korea, 2020–2024 (HIRA database).

Year	Total Costs, KRW (×1000)	Insurance-Covered, KRW (×1000)	Patient Copay, KRW (×1000)	Insurance Coverage, %
2020	4,250,352	3,741,956	508,396	88.0%
2021	4,141,246	3,610,123	531,123	87.2%
2022	4,260,066	3,758,447	501,619	88.2%
2023	5,130,399	4,497,214	633,185	87.7%
2024	4,790,086	4,210,872	579,214	87.9%

KRW = Korean Won; HIRA = Health Insurance Review and Assessment.

**Table 3:** Volume forecast for testicular cancer patients during 2026–2028: multi-method ensemble analysis.

Year	ARIMA(1,1,0)	Linear Regression	Holt–Winters Exp. Smoothing	Ensemble (Weighted)	Linear Regression 95% PI
2020–2024 (Observed)	2309–2992	—	—	—	—
2026	3617	3198	3005	3287	2856–3540
2027	4707	3356	3151	3767	2962–3749
2028	6604	3514	3297	4531	3066–3962

Ensemble weights: ARIMA(1,1,0) = 0.35; linear regression = 0.35; Holt–Winters = 0.30. The 95% prediction interval (PI) column reflects the linear regression component’s PI only and does not bound the ensemble point estimate, which incorporates the ARIMA component’s higher projections. Note: the 2027 and 2028 ensemble point estimates exceed the linear regression 95% PI upper bound, illustrating the substantial additional uncertainty introduced by the ARIMA component on a short series (*n* = 5); this divergence should be interpreted as a limitation of forecasting on five annual observations. ARIMA = autoregressive integrated moving average; PI = prediction interval; ensemble methodology after GBD 2019 [27].

Figure 1A shows the annual patient volume with the fitted linear trend line (R^2^ = 0.885, *p* = 0.017). The fitted regression slope corresponds to approximately 158 additional patients per year; the mean absolute annual increase between 2020 and 2024 was 171 patients per year. Figure 1B presents the three-method comparison for 2026–2028 forecasts alongside the ensemble estimate with linear regression 95% PI.

### 3.2. Testicular Cancer Forecast for 2026–2028

The exploratory ensemble forecast projected TC patient volume of 3287 (linear regression 95% PI: 2856–3540) in 2026, 3767 (linear regression 95% PI: 2962–3749) in 2027, and 4531 (linear regression 95% PI: 3066–3962) in 2028 (Table 3). It is noted that the 2027 and 2028 ensemble point estimates exceed the upper bound of the linear regression 95% PI; this occurs because the PI column reflects the linear regression component only, while the ensemble point estimate incorporates ARIMA-weighted projections that are substantially higher, as documented in the table. ARIMA(1,1,0) produced the highest estimates in 2027 and 2028 (4707 and 6604, respectively), reflecting strong upward momentum in the AR(1) component on a short series. Linear regression yielded the most conservative estimates (3198–3514), while Holt–Winters produced intermediate values (3005–3297). The wide divergence among methods highlights the substantial uncertainty inherent in forecasting from five annual observations; all projections should be treated as exploratory directional estimates for planning purposes only.

### 3.3. Urological and Oncological Co-Occurring Condition Trends, 2020–2024

**Figure 2:**
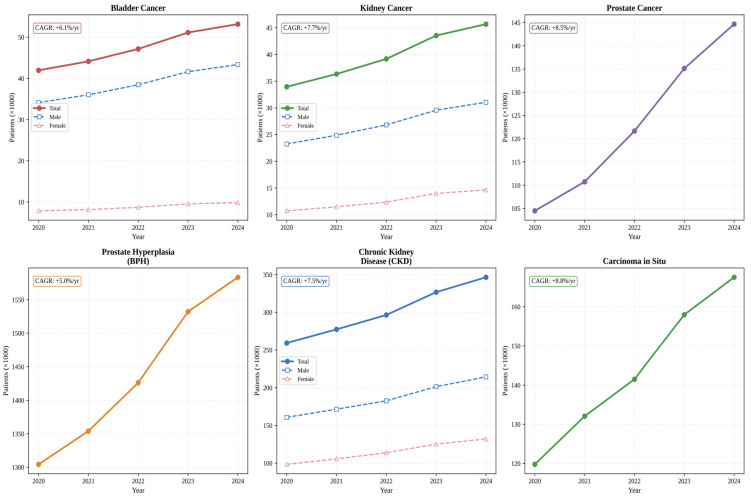
Longitudinal trends in urological and oncological co-occurring conditions, South Korea, 2020–2024 (HIRA database). CAGR = compound annual growth rate.

Nine of ten analyzed disease categories showed increasing patient volumes over 2020–2024 (Table 4; Figure 2); pyelonephritis was the exception, showing an overall negative CAGR of −1.37% with a transient decrease in 2021 before partially recovering to 224,570 in 2024, remaining below the 2020 baseline (237,301). Prostate cancer exhibited the highest absolute growth (104,483 → 144,661; +38.4%; CAGR: +8.5%), followed by kidney cancer (+34.5%; CAGR: +7.7%) and CKD (+33.7%; CAGR: +7.5%). Bladder cancer increased by 26.7% (CAGR: +6.1%). Cystitis (predominantly female, F:M ratio ≈ 15:1) and neurogenic bladder showed consistent increases of 8.8% and 24.2%, respectively. Pyelonephritis was the only condition with an overall negative CAGR (−1.37%), with a transient decrease in 2021, a partial recovery through 2023, and a slight decline in 2024; the 2024 patient count (224,570) remained below the 2020 baseline (237,301).

### 3.4. Sex-Stratified Analysis

**Figure 3:**
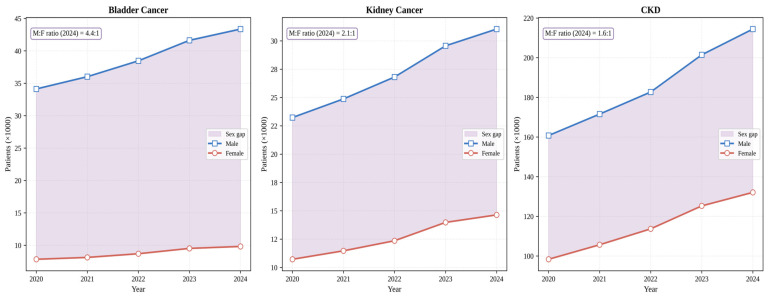
Sex-stratified patient trends for bladder cancer, kidney cancer, and chronic kidney disease (CKD), South Korea, 2020–2024. M:F = male-to-female patient ratio in 2024.

Sex-stratified analysis was performed for conditions with available sex-disaggregated data (Figure 3). For bladder cancer, the male-to-female (M:F) ratio was 4.4:1 in 2024 (males: 43,362; females: 9,820), stable throughout the study period. For kidney cancer, the M:F ratio was 2.1:1 in 2024 (males: 31,041; females: 14,637). CKD showed an M:F ratio of 1.6:1 in 2024 (males: 214,439; females: 132,079). All three conditions showed faster absolute growth in males than females over the study period.

### 3.5. Correlation Between Testicular Cancer and Co-Occurring Conditions

**Figure 4:**
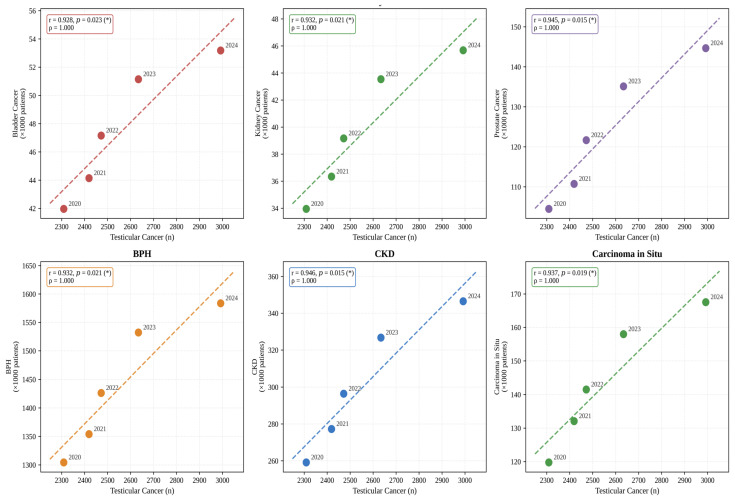
Scatter plots and regression lines for Pearson correlation between testicular cancer (TC) patient volume and six urological/oncological co-occurring conditions, South Korea, 2020–2024. Each panel includes the Pearson r, *p*-value, and significance indicator. Asterisks (*) denote statistical significance at *p* < 0.05.

All six analyzed co-occurring conditions demonstrated strong positive Pearson correlations with TC patient volume over 2020–2024 (Table 5; Figure 4). CKD showed the strongest correlation (r = 0.946, ρ = 1.000), followed by prostate cancer (r = 0.945, ρ = 1.000), kidney cancer (r = 0.932, ρ = 1.000), BPH (r = 0.932, ρ = 1.000), carcinoma in situ (r = 0.937, ρ = 1.000), and bladder cancer (r = 0.928, ρ = 1.000). All correlations exceeded r = 0.90; however, given *n* = 5 annual aggregate observations, these high correlations are expected from parallel secular upward trends across multiple conditions and do not provide evidence of individual-level co-occurrence or causal association. The *p*-values reported in Table 5 are highly unstable at *n* = 5 and are included for completeness only; all correlations should be interpreted as descriptive, exploratory trend associations susceptible to shared secular trend confounding.

### 3.6. Healthcare Cost and Utilization

**Figure 5:**
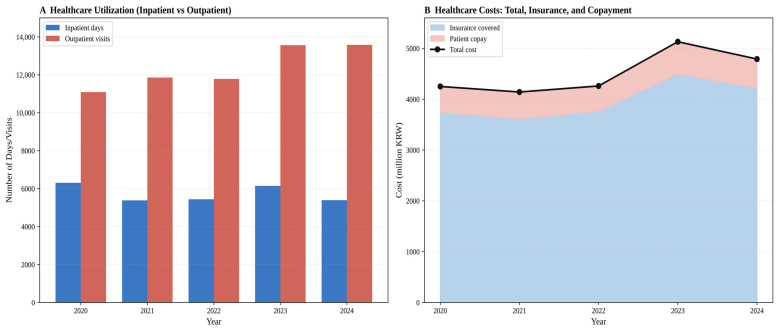
Healthcare utilization and cost trends for testicular cancer patients, South Korea, 2020–2024. (**A**) Inpatient days vs. outpatient visits. (**B**) Total healthcare costs stratified by insurance-covered and patient copayment components.

Total TC healthcare costs ranged from 4.14 billion KRW (2021) to 5.13 billion KRW (2023; Figure 5). Insurance-covered costs accounted for approximately 88% of total costs across all years. Total outpatient visit volume (all outpatient encounters, distinct from the annual unique outpatient patient counts reported in Table 1) grew substantially (+22.5%), from 11,087 in 2020 to 13,578 in 2024, while inpatient days remained relatively stable (range: 5380–6305). The cost peak in 2023 coincided with the highest patient volume that year (2634), possibly reflecting increased diagnostic workup and surgical intervention.

## 4. Discussion

This analysis of HIRA administrative claims data from 2020 to 2024 demonstrated a consistent and substantial increase in TC patient volume in South Korea (CAGR: 6.69%/year), with exploratory ensemble forecasts projecting continued growth through 2028. These findings are consistent with, and extend, the broader global and regional literature documenting rising TC patient volume in East Asia [1,2,3]. It should be noted that HIRA aggregate data represent annual unduplicated patient counts without population denominators or age-standardized rates; the observed trend reflects claims-based patient volume, which is influenced by population growth, ageing, and changes in healthcare utilization and cannot be equated with true incidence without further linkage to census denominator data.

The rising TC patient volume trend in South Korea may reflect multiple intersecting drivers. Improved detection through expanded health screening programs, increased public awareness of male urological health, and advances in urological imaging technology all increase case ascertainment rates without necessarily reflecting true incidence increases [4,7]. Additionally, epidemiological evidence from high-income countries implicates hormonal, environmental, and lifestyle factors—including exposure to endocrine-disrupting chemicals, sedentary behavior, and altered pubertal timing—in the secular rise in TC incidence [5,20]. Whether similar environmental determinants are operative in the Korean context warrants dedicated epidemiological investigation with individual-level data.

The exploratory correlations between TC patient volume and all six analyzed co-occurring conditions (r: 0.928–0.946) require cautious interpretation. Given *n* = 5 annual observations and universal upward secular trends across all analyzed conditions, near-unity correlations are an expected mathematical artifact of parallel trend confounding rather than evidence of biological or causal linkage between TC and the co-occurring conditions. The *p*-values associated with these correlations (all <0.05) are unreliable at *n* = 5 and are presented for descriptive completeness only. Population aging, improved detection, post-COVID-19 healthcare utilization rebound, and expanding HIRA coverage are all shared secular drivers that could produce these correlations independently of any TC-specific association [14,19]. The correlations must not be interpreted as evidence of causal association or individual-level co-occurring conditions. Nonetheless, the consistent co-occurrence of rising TC volume with rising prostate cancer, kidney cancer, CKD, and bladder cancer underscores the importance of integrated urological surveillance systems that can simultaneously monitor multiple conditions in high-risk male populations [8,9,10].

The exploratory 2026–2028 ensemble forecast projects continued TC growth, reaching an estimated 4531 patients by 2028 under a sustained linear-trend assumption. The wide divergence between ARIMA estimates (6604 in 2028) and linear regression estimates (3514) highlights the sensitivity of short-term forecasts to model assumptions when data series are short (*n* = 5); the ARIMA(1,1,0) model is particularly prone to producing extreme extrapolations on short series due to high leverage of the AR(1) coefficient. It is important to note that the 2027 and 2028 ensemble point estimates fall above the linear regression 95% PI upper bounds; this occurs because the PI column in Table 3 reflects the linear regression component only and does not account for the additional uncertainty introduced by ARIMA weighting. A fully specified ensemble PI bounding all component projections would be wider and should be the focus of future work with longer data series. These projections are best interpreted as directional planning estimates for urological oncology workforce and resource planning, not as precise predictions.

### 4.1. Future Research Directions

The present study is descriptive and ecological in design; it documents claims-based trends and exploratory aggregate-level associations but cannot identify the modifiable determinants of TC or establish causal relationships. Three priorities for future research are proposed to address these limitations and translate the present findings into actionable public health evidence.

First, systematic investigation of modifiable risk factors is required. Future studies should use standardized questionnaires, occupational exposure registries, and prospective cohort designs to assess lifestyle factors (sedentary behavior, dietary patterns, body mass index), environmental exposures (endocrine-disrupting chemicals, pesticides, heavy metals), occupational hazards, and socioeconomic determinants in the Korean context [5,20]. Risk factor surveys linked to HIRA or NHIS individual-level records would allow multivariable adjustment and identification of the factors most strongly associated with TC risk, providing the epidemiological foundation for evidence-based prevention programs.

Second, causal inference frameworks should be applied to move beyond the descriptive correlations reported here. The strong aggregate-level associations observed in the present study are susceptible to residual confounding and reverse causation, and neither causality nor directionality can be inferred from the current data. Two-sample Mendelian randomization (MR) using genetic variants as instrumental variables offers a particularly powerful approach for assessing causal relationships between candidate exposures and TC risk, as genetic instruments are not subject to the same confounding and reverse causation biases that affect observational studies [22]. Large-scale genome-wide association study (GWAS) summary statistics for TC and related traits are now publicly available, enabling two-sample MR designs that could, for example, evaluate whether height, inflammatory cytokine levels, or metabolite profiles causally influence TC risk—building on published MR work exploring these specific pathways [22,29]. Applying MR and related causal inference methods to TC in the Korean context, where genetic and environmental risk factor profiles may differ from European populations, represents a high-priority research agenda.

Third, causal evidence from MR and individual-level epidemiological studies should be complemented by natural experiments and quasi-experimental designs to evaluate the real-world impact of specific public health interventions on TC trends. Interrupted time-series analyses of HIRA claims data following policy changes (e.g., modifications to national cancer screening eligibility or occupational safety regulations) could provide policy-relevant causal estimates without requiring a randomized trial [30]. Together, these three research directions would move the field from descriptive epidemiology toward causally informed, actionable TC prevention strategies in South Korea.

### 4.2. Limitations

Several limitations must be acknowledged. First, and most fundamentally, HIRA aggregate data provide annual unduplicated patient counts without population denominators, age-stratified data, or a clearly defined incident-case definition. The term “incidence” has therefore been replaced throughout with “patient volume” to accurately reflect what the data capture; true incidence rates would require linkage to census population denominators and age standardization, which are not available in this aggregate dataset. Second, HIRA data represent administrative claims records, which may introduce coding-based misclassification bias in disease ascertainment. Third, the study period (2020–2024) spans the COVID-19 pandemic, which may have transiently suppressed healthcare utilization in 2020–2021 and subsequently driven a rebound effect, potentially inflating trend estimates. Fourth, the short time series (*n* = 5 annual data points) limits the statistical power of correlation analyses and the precision of ARIMA model identification; all *p*-values are unreliable at *n* = 5 and all forecasts should be treated as exploratory directional projections. The 2027 and 2028 ensemble point estimates falling outside the linear regression 95% PI reflect a limitation of the ensemble PI specification, which should be understood as bounding the linear regression component only; a fully specified ensemble PI encompassing ARIMA uncertainty would be substantially wider. Fifth, the correlation analyses are based on aggregate annual time-series data and are highly susceptible to common secular trend confounding; high Pearson r values near 1.0 are expected when multiple conditions share upward secular trends and do not constitute evidence of individual-level co-occurrence or causal association. Sixth, individual-level risk factor data (e.g., cryptorchidism history, family history, germ cell tumor histology) are not available in HIRA aggregate data, precluding adjustment for potential confounders. Seventh, TC is an exclusively male disease, but HIRA sex-stratified aggregate data for TC were not separately available for direct male-only rate calculation; total patient counts were used as a proxy.

## 5. Conclusions

Testicular cancer patient volume in South Korea increased by 29.6% over 2020–2024, with a CAGR of 6.69%/year, and exploratory ensemble forecasts project continued growth to approximately 3287–4531 patients annually by 2026–2028 under a sustained linear-trend assumption. Nine of ten analyzed urological and oncological conditions showed upward trends, and all six analyzed conditions demonstrated strong exploratory correlations with TC patient volume (r > 0.92) that reflect parallel secular trends in aggregate claims data rather than individual-level co-occurrence or causal association. These findings support claims-based trend monitoring, urological oncology resource planning, and hypotheses for future individual-level epidemiological research; individual-level NHIS or HIRA cohort data with population denominators, age standardization, and confounding adjustment are required before causal inference or screening policy recommendations can be made.

## Figures and Tables

**Table 4:** Urological and oncological co-occurring condition patient trends, South Korea, 2020–2024 (HIRA database).

Disease Category	2020	2021	2022	2023	2024	CAGR, %
Testicular cancer (ICD-10: C62)	2309	2419	2472	2634	2992	+6.69
Bladder cancer (C67)	41,966	44,145	47,159	51,147	53,182	+6.10
Kidney cancer (C64)	33,951	36,340	39,165	43,541	45,678	+7.69
Prostate cancer (C61)	104,483	110,736	121,665	135,119	144,661	+8.46
BPH (N40)	1,304,329	1,354,026	1,426,279	1,532,151	1,583,627	+4.99
CKD (N18)	259,116	277,252	296,397	326,736	346,518	+7.53
Cystitis (N30)	1,567,895	1,573,392	1,631,313	1,695,519	1,704,662	+2.12
Neurogenic bladder (N31)	563,736	620,329	643,274	688,598	700,361	+5.56
Pyelonephritis (N10–N12)	237,301	217,977	215,655	227,652	224,570	–1.37
Carcinoma in situ (D00–D09)	119,757	132,071	141,506	157,971	167,549	+8.78

BPH = benign prostatic hyperplasia; CAGR = compound annual growth rate; CKD = chronic kidney disease; HIRA = Health Insurance Review and Assessment; ICD-10 = International Classification of Diseases, 10th revision.

**Table 5:** Pearson and Spearman correlation coefficients between testicular cancer patient volume and urological/oncological co-occurring conditions, South Korea, 2020–2024 (*n* = 5).

Co-Occurring Condition	Pearson’s r	*p*-Value (r)	Spearman’s ρ	Interpretation
Chronic Kidney Disease (CKD)	0.946	<0.05	1.000	Very strong positive
Prostate Cancer	0.945	<0.05	1.000	Very strong positive
Carcinoma in Situ	0.937	<0.05	1.000	Very strong positive
Kidney Cancer	0.932	<0.05	1.000	Very strong positive
Benign Prostatic Hyperplasia	0.932	<0.05	1.000	Very strong positive
Bladder Cancer	0.928	<0.05	1.000	Very strong positive

*p*-values are approximate given *n* = 5 and are highly unstable; they are reported for completeness only and should not be used to draw inferential conclusions. All correlations are exploratory descriptive trend associations reflecting parallel secular upward trends in aggregate annual claims data; they do not establish individual-level co-occurrence or causal relationships and are susceptible to ecological fallacy. Very strong: |r| > 0.90. CKD = chronic kidney disease.

## Data Availability

The data presented in this study are publicly available from the Health Insurance Review and Assessment (HIRA) Open Data Portal at https://opendata.hira.or.kr (accessed on 20 March 2026). Aggregate-level disease burden statistics by year, sex, and visit type are openly accessible without registration. The processed dataset and analysis code used in this study are available from the corresponding author upon reasonable request.
